# The mitochondrial DNA control region shows genetically correlated levels of heteroplasmy in leukocytes of centenarians and their offspring

**DOI:** 10.1186/1471-2164-8-293

**Published:** 2007-08-29

**Authors:** Giuseppina Rose, Giuseppe Passarino, Vittorio Scornaienchi, Giuseppe Romeo, Serena Dato, Dina Bellizzi, Vincenzo Mari, Emidio Feraco, Raffaele Maletta, Amalia Bruni, Claudio Franceschi, Giovanna De Benedictis

**Affiliations:** 1 Department of Cell Biology, University of Calabria. 87036 Rende, Italy; 2 Italian National Research Center on Ageing (INRCA). 87100 Cosenza, Italy; 3 Regional Neurogenetic Center, ASL 6 Viale Perugini. 88046 Lamezia Terme, Italy; 4 Department of Experimental Pathology and Interdepartmental Center L. Galvani, University of Bologna, Bologna, Italy

## Abstract

**Background:**

Studies on heteroplasmy occurring in the mitochondrial DNA (mtDNA) control region (CR) in leukocytes of centenarians and younger subjects have shown that the C150T somatic transition is over-represented in centenarians. However, whether the occurrence/accumulation of heteroplasmy is a *phenotypic consequence *of extreme ageing or a *genetically controlled event *that may favor longevity is a question that deserves further attention. To clarify this point, we set up a Denaturing High Performance Liquid Chromatography (DHPLC) protocol to quantify mtDNA CR heteroplasmy. We then analyzed heteroplasmy in leukocytes of centenarians (100 subjects), their offspring and nieces/nephews (200 subjects, age-range 65–80 years, median age 70 years), and in leukocytes of 114 control subjects sex- and age-matched with the relatives of centenarians.

**Results:**

The centenarians and their descendants, despite the different ages, showed similar levels of heteroplasmy which were significantly higher than levels in controls. In addition we found that heteroplasmy levels were significantly correlated in parent-offspring pairs (r = 0.263; p = 0.009), but were independent of mtDNA inherited variability (haplogroup and sequence analyses).

**Conclusion:**

Our findings suggest that the high degree of heteroplasmy observed in centenarians is genetically controlled, and that such genetic control is independent of mtDNA variability and likely due to the nuclear genome.

## Background

Mitochondrial DNA (mtDNA) is much more exposed to mutagenic events than nuclear DNA (nDNA) due to its high replication rate, lack of histone-like proteins, scarcity of repair enzymes, and production of Reactive Oxygen Species (ROS) which results from Oxidative Phosphorylation (OXPHOS) in mitochondria. The fate of heteroplasmic mutations depends on several factors, including type and location of the variation, replication rate of the cell, and also to chance since the mutant molecules can be randomly lost as a consequence of mitochondria replicative segregation. In any case, since mutations are stochastic events, mtDNA heteroplasmy tends to increase with age. Usually, a low level of heteroplasmy does not impair mitochondrial function, but once the level of mutant mtDNA exceeds a certain threshold, OXPHOS dysfunction may arise [1,2]. The cell tries to cope with such a stressful condition by increasing OXPHOS, and therefore producing ROS, in a vicious circle that may become lethal to the cell itself.

As a rule, the age-related accumulation of mtDNA somatic mutations leads to a decline in mitochondrial function, which contributes to ageing and degenerative diseases [3,4]. In fact, most of the literature on mtDNA somatic mutations reports data on the role played by mtDNA heteroplasmy on age-related diseases, but recent findings open a new perspective. Zhang et al. [5] carried out a large-scale screening of the mtDNA main control region in leukocytes from centenarians and younger controls. They found that the C150T mutation is significantly more represented in centenarians than in younger controls, and provided evidence that somatic events, probably under nuclear genome control, contribute to the striking selective accumulation of this mutation in centenarians. In the same report, using fibroblast longitudinal studies the authors showed an age-related somatic expansion of the mutation up to homoplasmy. Finally, 5' end analysis of nascent heavy mtDNA strands revealed a new replication origin at position 149, substituting the one at 151, only in fibroblasts or immortalised lymphocytes carrying the C150T mutation. On the whole, the data showed that a high level of C150T heteroplasmy, possibly up to a new homoplasmy arrangement, might be favourable for longevity. In agreement, a significant association between the inherited C150T mutation and longevity has been observed in both Finnish and Japanese populations [6].

The study by Zhang et al. [5] is of great value because it indicates a possible beneficial effect on longevity by an mtDNA somatic mutation able to restore the mitochondrial replication machinery. Therefore, it seemed worthwhile to further investigate possible links between mtDNA CR heteroplasmy and longevity. Since longevity shows clear patterns of familiarity [7,8], the study of such a heteroplasmy in relatives of centenarians may help to clarify the role of mtDNA somatic variability in longevity.

We set up a Denaturing High Performance Liquid Chromatography (DHPLC) protocol by which the heteroplasmy of an mtDNA CR fragment encompassing the C150T mutation could be quantified. Then we compared the levels of heteroplasmy between relatives of centenarians (offspring and nieces/nephews) and age-matched controls. Indeed, if heteroplasmy accumulates because of age-related stochastic events, it should be similar between age-matched groups regardless of the genetic relationship with centenarians. On the contrary, if the heteroplasmy is under genetic control, it should be higher in relatives of centenarians than in controls. In this case, the heteroplasmy occurring in the mtDNA region under study may be regarded as a contributing factor to familial recurrence of longevity.

## Results

### DHPLC reference curve and sensitivity of the method

**Figure 1:**
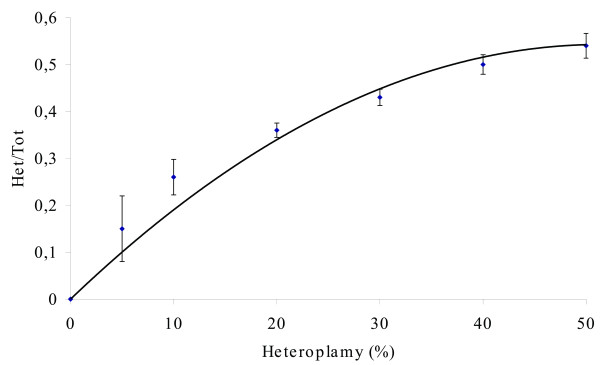
**DHPLC reference curve assembled from clones of mtDNA CR (16531 nt -261 nt) including either C150 or T150**. The two clones were combined to generate samples having heteroplasmy levels of 0%, 5%, 10%, 20%, 30%, 40%, 50 %. Het/Tot is the ratio between the height of the Heteroplasmic peak and the Total height of homoplasmic plus heteroplasmic peaks. Bars denote the standard deviation in triplicate experiments. The observed values were used to fit a 2^nd ^degree polynomial function y = β_1_x + β_2_x^2^.

In order to quantify the levels of heteroplasmy in the biological samples under study, we applied DHPLC to artificial heteroplasmic samples and assembled the curve shown in Fig. 1. The reference curve was used for estimating the levels of heteroplasmy in the biological samples.

In order to compare DHPLC and sequencing sensitivity in revealing heteroplasmy, we submitted all the artificial samples reported in Fig. 1 to sequence analysis. We found that DHPLC is able to reveal up to 5% of heteroplasmy, while the minimum level of heteroplasmy detectable by sequence analysis is roughly 20–25%.

Before applying DHPLC to the biological samples, we verified that the PCR conditions were specific for the mtDNA fragment under study (16531 nt-261 nt). No signal indicating DNA amplification was observed by applying our PCR protocol to DNA extracted from rho-zero cells (cells depleted of mitochondria). Furthermore, the PCR primers gave negative results to BLAST search.

### Heteroplasmy in families of centenarians

**Figure 2:**
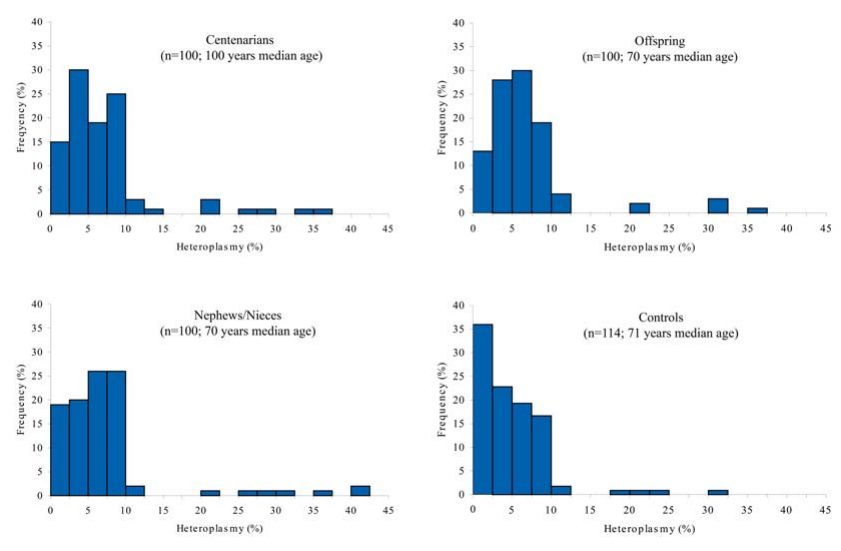
**Histograms showing mtDNA CR heteroplasmy in the four sample groups**. Heteroplasmy is estimated from the DHPLC reference curve reported in Fig.1.

DHPLC was then applied to PCR products of DNA extracted from leukocytes of four sample groups: centenarians, offspring and nephews/nieces of centenarians, and controls. Fig. 2 shows the distribution of the levels of heteroplasmy in the four groups, as estimated using the reference curve in Fig. 1.

Fig. 2 shows that approximately 15% of both centenarians and their children, approximately 20% of nephews/nieces of centenarians and more than 35% of controls display levels of heteroplasmy lower than 2.5%. Therefore, not only do rather few centenarians have low heteroplasmy, but the same occurs in their younger relatives.

We checked the statistical significance of the differences shown in Fig. 2 by comparing the patterns of heteroplasmy between pairs of samples. Centenarians differed from younger controls (p = 0.001) while they did not from their relatives (p = 0.699 and p = 0.944 by comparing centenarians with offspring and nieces/nephews, respectively). What is more, the comparison between age-matched groups revealed that heteroplasmy differed between groups according to the presence/absence of a centenarian in the family (p = 0.666 between offspring and nieces/nephews of centenarians; p = 0.006 between offspring of centenarians and controls; p = 0.003 between nieces/nephews of centenarians and controls). The above differences remained significant when the level of significance was reduced to α = 0.009 (six independent comparisons).

**Figure 3:**
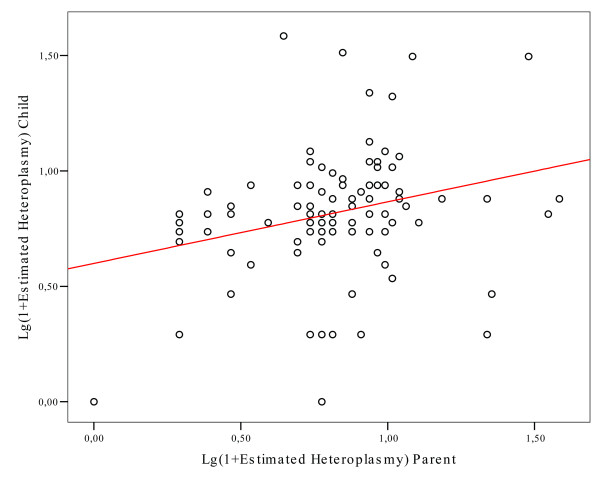
**Linear regression between heteroplasmy levels in centenarians (x axis) and their offspring (y axis)**. Log transformed values were used in order normalise the distribution. The regression line showed r = 0.263 (p = 0.009).

The finding that the centenarians' offspring displayed a heteroplasmic pattern similar to that of their very old parents suggested that the level of mtDNA CR heteroplasmy could be under genetic control. By linear regression analysis we confirmed a genetic control on this trait, as the levels of heteroplasmy resulted significantly correlated in parent-offspring pairs (Fig. 3; r = 0.263; p = 0.009).

**Figure 4:**
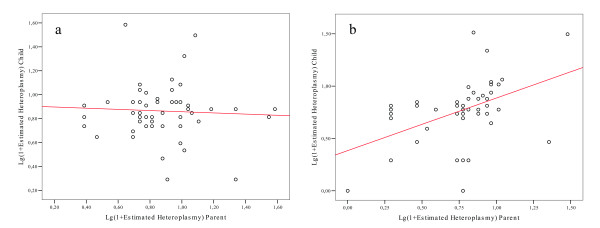
**Linear regression of heteroplasmy levels in parent-offspring pairs according to the sex of the parent: (a) male centenarian parents; (b) female centenarian parents**. The regression lines showed r = -0.053 (p = 0.704) and r = 0.456 (p = 0.001) in (a) and (b), respectively.

Which genome (mtDNA or nDNA) could account for the above results? In order to answer this question we partitioned the group of parents according to sex (Fig. 4), and found that the levels of heteroplasmy were significantly correlated in mother-offspring pairs (r = 0.456; p = 0.001) while there was no correlation in father-offspring pairs (r = -0.053; p = 0.704).

The above result, which indicated a maternal genetic control, prompted us to search for a possible association between inherited and epigenetic (somatic) mtDNA variability. Thus, we screened haplogroup and haplotype variability in both centenarians and controls, and analyzed the distribution of heteroplasmic subjects within each haplogroup/haplotype category. The data are reported in Tables 1 and 2.

**Table 1:** Inherited and epigenetic mtDNA variability. The number of subjects classified within a specific haplogroup is reported together with the number of subjects showing heteroplasmy levels higher than 5%. MtDNA haplogroups are classified according to Torroni et al., [27].

MtDNA Haplogroup	Centenarians		Controls	
	Absolute frequency	Heteroplasmic subjects	Absolute frequency	Heteroplasmic subjects

H	35	15	34	10
I	2	2	0	0
J	11	8	17	7
K	10	6	16	6
T	6	3	10	3
U	13	7	11	4
V	1	1	1	0
W	2	1	4	3
X	9	8	5	3
Other	11	4	16	6

Total	100	55	114	47

**Table 2:** Variant sites found in mtDNA CR (16531 nt – 261 nt). For each haplotype the absolute frequency in centenarians (A) and controls (B) is reported. The number of subjects showing DHPLC heteroplasmy levels higher than 5% is reported in parenthesis. rCRS refers to the revised Cambridge Reference Sequence [28].

	Nt	20	55	57	64	72	73	93	114	143	146	150	151	152	153	182	185	188	189	191	194	195	198	199	200	204	207	217	225	226	227	228	235	239
	rCRS	A	T	T	C	T	A	A	C	G	T	C	C	T	A	C	G	A	A	A	C	T	C	T	A	T	G	T	G	T	A	G	A	T

A	B																																	
27(14)	18(3)	-	-	-	-	-	G	-	-	-	-	-	-	-	-	-	-	-	-	-	-	-	-	-	-	-	-	-	-	-	-	-	-	-
2 (1)	0	-	-	-	-	C	-	-	-	-	-	-	-	-	-	-	-	-	-	-	-	-	-	-	-	-	-	-	-	-	-	-	-	-
3 (3)	0	-	-	-	-	-	G	-	-	-	-	-	-	-	-	-	A	G	-	-	-	-	-	-	-	-	-	-	-	-	-	A	-	-
21(8)	24(6)	-	-	-	-	-	-	-	-	-	-	-	-	-	-	-	-	-	-	-	-	-	-	-	-	-	-	-	-	-	-	-	-	-
5 (3)	7 (4)	-	-	-	-	-	-	-	-	-	-	-	-	C	-	-	-	-	-	-	-	-	-	-	-	-	-	-	-	-	-	-	-	-
1 (0)	0	-	-	-	-	-	-	G	-	-	-	-	-	-	-	-	-	-	-	-	-	C	-	-	-	-	-	-	-	-	-	-	-	-
1 (1)	2 (0)	-	-	-	-	-	G	-	-	-	-	T	-	-	-	-	-	-	-	-	-	C	-	-	-	-	-	-	-	-	-	-	-	-
1 (1)	0	-	-	-	-	-	-	-	-	-	-	-	-	-	-	-	-	-	-	-	-	-	-	-	-	-	-	-	-	-	-	-	G	-
1 (1)	0	-	-	-	-	-	G	-	T	-	-	-	-	-	-	-	-	-	-	-	-	-	-	-	-	-	-	-	-	-	-	-	-	-
1 (1)	7 (6)	-	-	-	-	-	G	-	-	-	-	-	-	C	-	-	-	-	-	-	-	-	-	-	-	-	-	-	-	-	-	-	-	-
1 (1)	0	-	-	-	-	-	G	-	-	-	-	T	-	-	-	-	-	-	G	-	-	-	-	-	-	-	-	-	-	-	-	-	-	-
1 (1)	0	-	-	-	-	-	G	-	-	-	C	-	-	-	G	-	-	-	-	-	-	C	-	-	-	-	-	-	A	C	-	-	-	-
4 (4)	7 (1)	-	-	-	-	-	G	-	-	-	-	-	-	-	-	-	-	-	-	-	-	C	-	-	-	-	-	-	-	-	-	-	-	-
1 (1)	0	-	-	-	-	-	G	-	-	A	-	-	-	-	-	-	-	-	-	-	-	C	-	-	-	-	-	-	A	C	-	-	G	-
1 (1)	2 (0)	-	-	-	-	-	G	-	-	-	-	T	-	-	-	-	-	-	-	-	-	-	-	-	-	-	-	-	-	-	-	-	-	-
1 (0)	0	-	-	-	-	-	-	-	-	-	-	-	-	-	-	-	-	-	-	-	-	-	-	-	-	-	-	-	-	-	-	A	-	-
2 (2)	0	-	-	-	-	-	G	-	-	-	-	-	T	C	-	-	-	-	-	-	-	-	-	-	-	-	-	-	-	-	-	-	-	-
3 (1)	2 (2)	-	-	-	-	-	G	-	-	-	-	-	-	-	-	-	A	-	-	-	-	-	-	-	-	-	-	-	-	-	-	A	-	-
1 (0)	0	-	-	-	-	-	-	-	-	-	-	-	-	-	-	-	-	-	G	-	-	-	-	-	-	-	-	-	-	-	-	-	-	-
1 (0)	0	-	-	-	-	-	G	-	-	-	-	-	-	-	G	-	-	-	-	-	-	C	-	-	-	-	-	-	A	C	-	-	-	-
1 (0)	0	-	-	-	-	-	G	-	-	A	C	-	-	C	-	-	-	-	-	-	-	C	-	-	-	-	-	-	-	-	-	-	-	-
2 (1)	0	-	-	-	-	-	-	-	-	-	-	-	-	-	-	-	-	-	-	-	-	-	-	-	G	-	-	-	-	-	-	-	-	-
1 (0)	0	-	-	-	-	-	G	-	-	-	C	-	-	C	-	-	-	-	-	-	-	-	-	-	-	-	-	C	-	-	-	-	-	-
1 (1)	6 (5)	-	-	-	-	-	G	-	-	-	-	T	-	C	-	-	-	-	-	-	-	-	-	-	-	-	-	-	-	-	-	-	-	-
1 (1)	0	-	-	-	T	-	G	-	-	-	-	-	-	-	G	-	-	-	-	-	-	C	-	-	-	-	-	-	A	C	-	-	-	-
1 (0)	0	-	C	C	-	-	-	-	-	-	-	-	-	-	-	-	-	-	-	-	-	C	-	-	-	-	-	-	-	-	-	-	-	-
2 (1)	0	-	-	-	-	-	-	-	-	-	-	-	T	-	-	-	-	-	-	-	-	-	-	-	-	-	-	-	-	-	-	-	-	C
2 (0)	7 (1)	-	-	-	-	-	-	-	-	-	C	-	-	-	-	-	-	-	-	-	-	-	-	-	-	-	-	-	-	-	-	-	-	-
1 (1)	0	-	-	-	-	-	-	-	-	-	-	T	-	-	-	-	-	-	-	-	-	-	-	-	-	-	-	-	-	-	-	-	-	-
1 (0)	0	-	-	-	T	-	-	-	-	-	-	-	-	-	-	-	-	-	-	-	-	C	-	-	-	-	-	-	-	-	-	-	-	-
1 (1)	0	-	-	-	-	-	G	-	-	-	C	-	-	-	G	-	-	-	-	-	-	C	-	-	-	-	-	-	A	-	G	-	-	-
1 (1)	2 (2)	-	-	-	-	-	G	-	-	-	-	-	-	C	-	-	-	-		-	-	C	-	-	-	-	-	-	-	-	-	-	-	-
1 (1)	0	-	-	-	-	-	G	-	-	-	-	-	-	-	-	-	-	-	G	-	-	C	-	-	-	C	A	-	-	-	-	-	-	-
1 (1)	0	-	-	-	-	C	-	G	-	-	-	T	-	-	-	-	-	-	-	-	-	C	T	-	-	-	-	-	-	-	-	-	-	-
1 (1)	4 (2)	-	-	-	-	-	G	-	-	-	C	T	-	-	-	-	-	-	-	-	-	-	-	-	-	-	-	-	-	-	-	-	-	-
1 (1)	0	-	-	-	-	-	G	-	-	-	-	-	-	-	-	T	-	-	-	-	-	-	-	-	-	-	-	-	-	-	-	-	-	-
1 (0)	1 (0)	-	-	-	-	-	G	-	-	-	C	-	-	-	-	-	-	-	-	-	-	-	-	-	-	-	-	-	-	-	-	-	-	-
1 (1)	0	-	-	-	-	-	G	-	-	-	-	T	-	-	-	-	-	-	-	-	-	C	-	-	G	-	-	-	-	-	-	-	-	-
0	2 (1)	-	-	-	-	C	-	-	-	-	-	-	T	-	-	-	-	-	-	-	-	-	-	C	-	-	-	-	-	-	-	-	-	-
0	1 (0)	-	-	-	-	-	G	-	-	-	-	-	-	-	-	-	A	-	G	-	-	-	-	-	-	-	-	-	-	-	-	A	-	-
0	1 (0)	-	-	-	-	C	-	-	-	-	-	-	T	-	-	-	-	-	-	-	-	-	-	-	-	-	-	-	-	-	-	-	-	-
0	3 (3)	-	-	-	-	-	G	-	-	-	-	-	-	C	-	-	A	G	-	-	-	-	-	-	-	-	-	-	-	-	-	-	-	-
0	1 (1)	-	-	-	-	-	-	-	-	-	C	-	-	C	-	-	-	-	-	-	-	-	-	-	-	-	-	-	-	-	-	-	-	-
0	1 (1)	-	-	-	-	-	-	G	-	-	-	-	-	-	-	-	-	-	-	-	-	-	-	-	-	-	-	-	-	-	-	-	-	-
0	1 (1)	T	-	-	-	-	-	-	-	-	-	-	-	-	-	-	-	-	-	-	-	-	-	-	-	-	-	-	-	-	-	-	-	-
0	5 (2)	-	-	-	-	-	G	-	-	-	C	-	-	-	-	-	-	-	-	-	-	C	-	-	-	-	-	-	-	-	-	-	-	-
0	2 (1)	-	-	-	-	-	G	-	-	-	C	-	-	C	-	-	-	-	-	-	-	-	-	-	-	-	-	-	-	-	-	-	-	-
0	1 (0)	-	-	-	T	C	-	-	-	-	-	-	-	-	-	-	-	-	-	-	-	C	-	-	-	-	-	-	-	-	-	-	-	-
0	1 (0)	-	-	-	-	-	G	-	-	-	-	-	-	-	-	-	A	-	-	G	-	-	-	-	-	-	-	-	-	-	-	A	-	-
0	1 (0)	-	-	-	-	C	-	-	-	-	-	-	-	-	-	-	-	-	-	-	T	-	-	-	-	-	-	-	-	-	-	-	-	-
0	2 (2)	-	-	-	-	-	G	-	-	-	-	T	-	C	-	-	-	-	-	-	-	C	-	-	-	-	-	-	-	-	-	-	-	-
0	1 (1)	-	-	-	-	-	G	-	-	-	-	-	-	-	-	-	-	-	G	-	T	C	-	-	-	-	-	-	-	-	-	-	-	-
0	1 (1)	-	-	-	-	-	G	-	-	-	-	-	-	-	-	-	-	-	G	-	-	-	-	-	-	-	-	-	-	-	-	-	-	-
0	1 (1)	-	-	-	-	-	G	-	-	-	C	T	-	C	-	-	-	-	-	-	-	-	-	-	-	-	-	-	-	-	-	-	-	-

By comparing the distribution of heteroplasmic subjects within haplogroups (Table 1) or haplotypes (Table 2) with the distribution expected under random association between epigenetic and inherited variability, no significant difference was found either in centenarians or in controls (p > 0.2 by permutation tests).

In addition, we compared the pools of haplotypes (Table 2) between centenarians and controls by considering as inherited the variant whose sequence peak was higher than 80%. No significant difference was evident between the groups (p = 0.999 by permutation test); therefore the high heteroplasmy observed in centenarians was not due to one or few haplotypes which could be particularly prone to somatic mutations. On the whole, haplogroup and haplotype analyses consistently indicated that mtDNA CR heteroplasmy is independent of mtDNA inherited variability.

### The C150T somatic mutation

**Figure 5:**
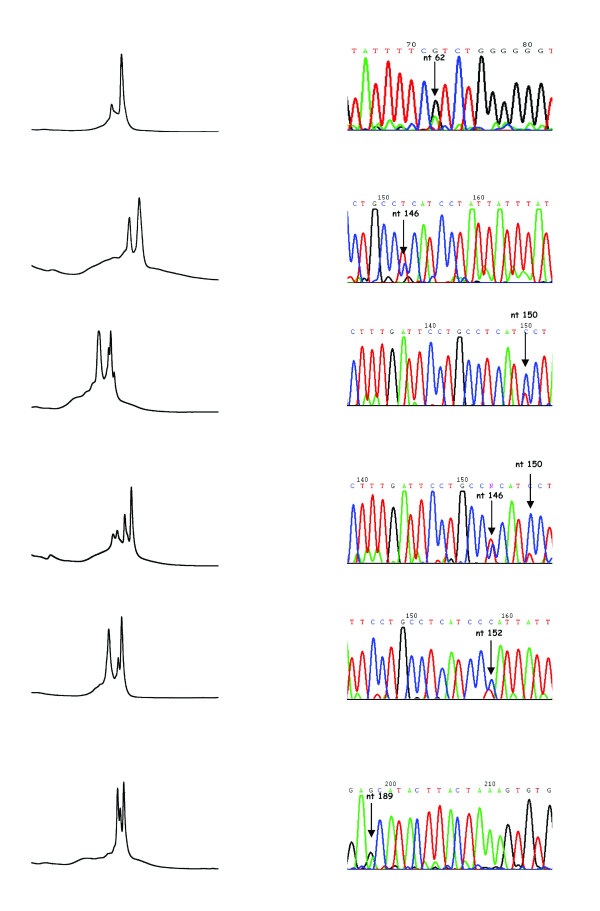
**DHPLC profiles observed in the samples having levels of heteroplasmy equal to or higher than 25%**. The sequence which characterizes each profile is shown on the right.

The DHPLC protocol used for assembling the reference curve of Fig. 1 had been set up using mtDNA cloned fragments that only differed in the C150T position. However, when we deal with biological samples, we cannot exclude that the DHPLC patterns of heteroplasmy we obtain might be due to other heteroplasmic sites. To highlight this point, first of all we carried out a careful visual inspection of every DHPLC profile in centenarians, their offspring, and controls. More than 60% of the samples showed DHPLC profiles comparable with those obtained with the different mixtures of C150 and T150 clones; however other profiles were also observed, suggesting the presence of additional heteroplasmic mutations in the region under study. Thus we compared DHPLC profile and sequence data whenever possible (requirement: minimum of 25% heteroplasmy). Out of 16 samples satisfying the requirement, we identified the six profiles reported in Fig. 5, where the corresponding heteroplasmic mutations detected by sequence analysis are also shown. Further data on sequence analyses on DHPLC fractions collected with a fraction collector are ongoing at present.

Table 3 summarises the proportion of different mutations found in centenarians, their offspring and controls. The C150T somatic mutation was present in 4 out of 7 centenarians, 4 out of 6 children of centenarians, and, in association with the T146C mutation, in 2 out of 3 controls.

**Table 3:** Heteroplasmic sites identified by sequencing mtDNA CR (16531 nt_261 nt) in subjects showing DHPLC heteroplasmy levels equal to or higher than 25%.

	Mutations				
	G62A	T146C	C150T	T152C	G189A

Centenarians	1	1	4		1
Offspring of centenarians		1	4^#^	1	
Controls		2*		1	

# One of the 4 children showing C150T heteroplasmy was the son of one of the 4 centenarians displaying C150T heteroplasmy.

* Subjects showing heteroplasmy for both T146C and C150T mutations.

## Discussion

The aim of the present study was to reconsider an intriguing observation: a strikingly high frequency of the C150T mutation in mtDNA CR in centenarians [5]. The observation was at odds with the consolidated idea that heteroplasmy is detrimental for attaining longevity, considering that a variety of mtDNA deletions and mutations accumulate with age [9] and that mitochondrial function declines with age [10]. The novelty of our approach was to compare mtDNA CR heteroplasmy in descendants of centenarians (both offspring and nephews/nieces) and age-matched controls unrelated to centenarians. The analysis of offspring of centenarians is a valuable tool in searching for susceptibility genetic factors in longevity [11]; however, it has never been used to investigate a putative role of mtDNA somatic variability on longevity.

The first requirement for our study was to set up a fast and reliable method to screen mtDNA heteroplasmy, and DHPLC met our requirement. Till now DHPLC has been applied to detect mutations on the entire mtDNA molecule in samples of rather limited size [12-16]. In our case, we needed to carry out quantitative comparisons of the level of heteroplasmy in a sole mtDNA region, the control region, but in a large population sample (414 subjects in total). The DHPLC protocol we set up provided reliable results (see standard deviations of the reference curve in Fig. 1) and was reasonably sensitive.

A further critical point was to establish that the PCR protocol was specific for the amplification of the 16531 nt-261 nt mtDNA fragment. In fact, it was recently shown that the pseudo-mitochondrial genome can induce errors in heteroplasmy interpretation [17]. The negative results we obtained both by processing rho-zero cells and by a BLAST search excluded that nuclear pseudogenes contaminated mtDNA PCR amplifications.

The most important finding presented here is that the patterns of mtDNA CR heteroplasmy do not differ between centenarians and their descendants, but differ between relatives of centenarians and age-matched controls (Fig. 2). This result ruled out that heteroplasmy was exclusively due to age-related stochastic mutations, however indicated that it was genetically controlled. In agreement, the levels of heteroplasmy were significantly correlated in parent-offspring pairs (Fig. 3). The observation that the correlation was significant in mother-offspring pairs while not in father-offspring pairs (Fig, 4a and 4b) suggested that a possible genetic control on heteroplasmy was due to the mitochondrial genome. However, other clues indicated a different, and probably more complicated, genetic control pattern. First, the lack of association between mtDNA haplotypes and heteroplasmy (Tables 2 and 3) rules out that there are mtDNA molecules more prone than others to somatic mutations. Second, both the tissue specificity of mtDNA CR point mutations [18] and the concordance of heteroplasmy higher in monozygotic than in dizygotic twins [5] denote that heteroplasmy is not related to the mtDNA haplotype the offspring inherited from the mother. Thus, although the genetic mechanism modulating the occurrence/accumulation of the mtDNA CR heteroplasmy needs further work to be elucidated, all the data suggest the involvement of nuclear sex specific factors.

It should also be noted that the heteroplasmy revealed by DHPLC does not refer to the sole C150T variability, but to additional possible mutations occurring in the entire 16531 nt-261 nt mtDNA fragment. In fact, the C150T mutation was found to be present in 10 out of 16 subjects (Table 3), while the remaining subjects showed other heteroplasmic mutations [5]. Interestingly, most of the observed heteroplasmic positions were either replication origins (position 146, see Ref. [19]) or contiguous to replication origins (positions 150 and 152 that flank the 151 replication origin; position 189 which is 2 bp from the 191 replication origin). Since the C150T transition is able to provide alternative replication origins [5], a similar effect could be hypothesized for the other mutations.

The results reported in Fig. 2, show that mtDNA CR heteroplasmy cannot be accounted for only by to age-related stochastic mutations. What is more, the finding that mtDNA CR heteroplasmy is greater in descendants of centenarians than in age-matched controls suggests a beneficial role of mtDNA heteroplasmy for attaining longevity. In fact, several data show that the offspring of centenarians have a better chance to attain longevity than the general population [20,21]. How could this apparent paradox be explained from a biological point of view? The well known mitochondrial theory of ageing proposes that age-associated mitochondrial dysfunction is a consequence of age-associated accumulation of somatic mutations in the mtDNA population. However, recent findings suggest that at least some aspects of the above theory require reconsideration [22]. In fact, a key for explaining the paradox that mtDNA heteroplasmy could be beneficial for longevity may be the new emerging concept of mitochondria complementation, which suggests that human cells are protected from mitochondrial dysfunction by complementation of mtDNA products in fused mitochondria [23]. The beneficial effect of complementation may be enhanced by efficient mtDNA replication, as provided by CR mutations which introduce alternative replication sites. In fact, multiple replication origins falling in this DLoop region could play a major role in accelerating mtDNA synthesis to satisfy developmental, physiological, or aging-related demands [19]. However, neither the replicative advantage of some variants nor the mitochondrial complementation can explain, by themselves, the heteroplasmy patterns of Fig. 2. By contrast, it is likely that the interplay among new replication origins, mitochondrial complementation and nuclear factors might provide an advantage for pursuing longevity by counteracting age-related mitochondrial damages. In this frame, the subjects who are genetically predisposed to mtDNA CR heteroplasmy would be clearly favoured in the demographic selection as defined by Perls et al. [24].

## Conclusion

By studying offspring and nephews/nieces of centenarians we have shown that mtDNA CR heteroplasmy is genetically controlled and it recurs in families of centenarians. This observation suggests a beneficial role of mtDNA heteroplasmy for attaining longevity and it may provide indirect evidence to the complementation of mitochondria in coping with age-related mitochondrial dysfunction.

## Methods

### Biological samples

A total of 414 subjects were analyzed: 100 trios composed of one centenarian, his/her child, his/her nephew/niece *plus *a control group of 114 unrelated subjects with no centenarian in the family. The age range in each of the four sample groups was as follows: 100–108 years in centenarians (median age 100 years, 53 males and 47 females); 65–80 years in offspring of centenarians (median age 70 years; 42 males and 58 females) as well as in nephews/nieces of centenarians (median age 70 years, 51 males and 49 females) and in the control group (median age 71 years, 50 males and 64 females). All subjects lived in Calabria (southern Italy) and their Calabrian ancestry was ascertained up to the grandparents' generation. The sampling was carried out in the frame of the ECHA research project [25]. All the subjects provided written informed consent for the use of their phenotypic and genetic data in studies on human ageing.

### Molecular analyses

Total DNA was extracted from blood buffy-coats following standard procedures.

### 1. PCR amplification

A 300 bp region of mtDNA encompassing the C150T site (region 16531-261) was amplified by 5'-AATAGCCCACACGTTCCCCTTA-3' forward primer and 5'-GCTGTGCAGACATTCAATTG-3' reverse primer (0,4 μM each) in a final volume of 25 μl, containing 100 ng DNA, 1.5 mmol/L MgCl_2_, 200 μmol/L of each dNTP, and 1 U of EuroTaq DNA polymerase (EuroClone). Amplification was performed in a Perkin Elmer Cetus 9600 PCR system. The amplification conditions were as follows: initial denaturation at 93°C for 30s, followed by 35 cycles at 93°C for 15s, 64°C for 20s, 72°C for 1 m. PCR products were checked by 2% agarose gel electrophoresis in TBE buffer with ethidium bromide staining.

### 2. DHPLC

After PCR fragments had been denatured for 3 m at 95°C, and gradually re-annealed from 95°C to 65°C in 30 m, 15 μl of each sample were injected onto a DNASep™ column of a Transgenomic Wave Nucleic Acid Fragment Analysis System (Transgenomic, San Jose, CA). The amplicons were eluted in 0.1 M triethylammonium acetate, pH 7, with a linear acetonitrile gradient at a flow rate of 0.9 ml/min. Temperature conditions were chosen according to the online program provided by Stanford University [26]. Mismatches were recognised by the appearance of two or more peaks in the elution profiles. The heights of DHPLC peaks were measured by using WAVEMAKER 4.0 software (Transgenomic San Jose).

### 3. Quantification of heteroplasmy levels

PCR products containing common (C150) and mutant (150T) sequences were cloned into a plasmid vector pGEM-T Easy by using the TA cloning kit (Invitrogen, USA) according to the manufacturer's protocol. The correct insertion of the PCR product was verified by sequence analysis.

In order to build a reference curve for measuring the levels of heteroplasmy in the biological samples, plasmids containing the common (C150) and the mutant (150T) sequences were mixed in different proportions (0% C with 100% T; 5% C with 95% T; 10% C with 90% T; 20% C with 80% T; 30% C with 70% T; 40% C with 60% T; 50% C with 50% T) and again subjected to PCR amplification. Using this approach, artificial samples having controlled conditions of C150T heteroplasmy were created. These samples were then subjected to DHPLC and a reference curve was assembled where the ratio between the height of the heteroduplex peak and that of the total peak was reported as a function of heteroplasmy, which varied according to the proportion between the two categories of cloned plasmids. The levels of heteroplasmy in the biological samples were then estimated on the reference curve.

### 4. Sequencing

PCR-amplified fragments were purified by QIAquick PCR purification Kit (Qiagen), and sequenced by fluorescence-based automated direct sequencing using a BigDye Terminator Cycle Sequencing Ready Reaction Kit in a 310 DNA sequencer (PE Applied Biosystems). Sequencing reaction mixtures contained 4 μl of Terminator Ready Reaction Mix, 200 ng of template, 3.2 pmol of each primer in a total volume of 20 μl. Cycle sequencing was carried out for 25 cycles at 96°C for 10s, 50°C for 5s, 60°C for 4 m in GeneAmp PCR system 9600. The extension products were purified using Centri-Sep™ spin columns (Princeton Separations).

### Statistical analysis

SPSS v.10 software (SPSS Inc., Chicago, IL, USA) was used for statistical analysis. Non-parametric two-sided Mann-Whitney-U test was used to verify if the patterns of heteroplasmy were different between sample groups. The level of significance was adjusted to α = 1-0,95^1/n^, where *n *represents the number of independent comparisons.

Permutation tests were used to verify if the population pools of mtDNA haplotypic sequences (region 16531-261) differed between the sample of centenarians and that of younger controls with no centenarian in the family.

## Authors' contributions

GR, GP, VS and RM : DHPLC analyses; DB: cloning. VM, EF and AB: sampling. VS, GR and SD: mtDNA genotyping. GP, GR, CF and GBB: work hypothesis, study design, and coordination. All the authors discussed the data and participated to the draft of the manuscript, which was finalized by GP and GDB.
